# ZnO doped C: Facile synthesis, characterization and photocatalytic degradation of dyes

**DOI:** 10.1038/s41598-023-41106-4

**Published:** 2023-08-30

**Authors:** Nasser Mohammed Hosny, Islam Gomaa, Maryam G. Elmahgary, Medhat A. Ibrahim

**Affiliations:** 1 Chemistry Department , Faculty of Science, Port Said University, POB 42522, Port Said, Egypt; 2 Nanotechnology Research Centre (NTRC), The British University in Egypt (BUE), Suez Desert Road, El Sherouk City, Cairo, 11837 Egypt; 3 Chemical Engineering Department, The British University in Egypt (BUE), El Shrouk City, Cairo, Egypt; 4 Department of Chemical Engineering, Massachusetts Institute of Technology, 77 Massachusetts Avenue, Cambridge, MA 02139 USA; 5 Spectroscopy Department, National Research Centre, 33 El-Bohouth St., Dokki, Giza, 12622 Egypt; 6 Molecular Modeling and Spectroscopy Laboratory, Centre for Excellence for Advanced Science, National Research Centre, 33 El-Bohouth St, Dokki, Giza, 12622, Egypt

**Keywords:** Environmental sciences, Materials science

## Abstract

Carbon doped ZnO nanoparticles have been synthesized from the thermal decomposition of Zinc citrate precursor. The precursor was synthesized from semi-solid paste and then subjected to calcination at 700 °C to produce ZnO nanoparticles. The precursor and ZnO were characterized by Fourier Transform Infrared Spectroscopy, UV–visible (UV–Vis) spectra, Transmission Electron Microscope, Field Emission Scanning Electron Microscope, Energy Dispersive Analysis by X-ray (EDAX), X-ray powder diffraction (XRD), and X-ray photoelectron spectroscopy (XPS). The results ensured the formation of hexagonal 2D-ZnO nanoparticles with a layer thickness of 25 nm. The optical band gap of ZnO was determined and found to be 2.9 eV, which is lower than the bulk. Photocatalytic degradation of Fluorescein dye as an anionic dye and Rhodamine B as a cationic dye was evaluated via C-ZnO NPs under UV irradiation. ZnO displayed 99% degradation of Fluorescein dye after 240 min and a complete photocatalytic degradation of Rhodamine B dye after 120 min under UV irradiation.

## Introduction

The discharge of industrial wastewater contaminated with organic dyes resulted from the processing of fabrics, pharmaceutics, cosmetics, and others, has become the main cause of excessive water contamination^1^. The exposure of dyes even in a small concentration can critically influence the water quality of the aquatic environment^2^. Dyes as Rhodamine B, and methylene blue are non-biodegradable, toxic and carcinogenic hazardous dyes^3, 4^. Fluorescein is a highly fluorescent dye that can be used to visualize the structure of materials and track the flow of fluids and stable over a wide range of pH and temperature conditions^5^ . Non-biodegradable and resistant dyes represent a big problem because they can persist in the environment for long periods of time, where they can have many of negative environmental impacts^6^. It need multiple processes, such as adsorption^7^, filtration^8^, and photocatalysis^9^, for efficient purification of water. Photo catalysis is considered an eco-friendly sustainable technique for the removal of dyes from wastewater^10, 11^. Photo catalysis is a promising approach for future techniques that rely on a renewable available and an inexpensive natural sunlight radiations^12, 13^. Nanostructure defects are critical in defining the properties and performance of nanostructures in the targeted applications^14^. Without enforced parameter like pH and Temperature few of photocatlysts have an efficient degradation impact of both anionic and cationic dyes^15, 16^. Two-dimensional materials are sheet-like nanomaterials that are made of thin multiple layers with a thickness of several nanometers^17, 18^. Nano-diameter materials have attracted increasing attention for photocatalytic applications over other morphologies because of their unique thickness and their doubly exposed active surface, peculiar nature of the electronic density of state spectrum^19^. The photocatalytic reactions depend on induction by UV–visible light lies on a surface of a semiconductor such as ZnO^20^. It is an excellent n-type semi-conductor with band gap energy (3.3 eV) it has unique characteristics as high photosensitivity, good physical and chemical stability and high electron mobility^17, 21, 22^. ZnO has significant potential as a powerful antibacterial agent and high safety profile that might eventually replace antibiotics^23^. These characteristic properties enabled ZnO to be a promising material for a variety of applications, as solar cells, photo-catalysis and gas sensor^24^.Metallic^25^ and non-Metallic (e.g. Carbon)^26^ doping has a significant impact on band gap engineering and photo-catalysis efficiency^27, 28^. The enhancement of photocatalytic efficiency for ZnO-carbon doped might be due to the good dye adsorption capacity, direct photo-oxidation of dye, and inhibition of photo-induced electron–hole recombination^29^. Doping synthesis usually need sophisticated methods lacking simplicity and high yield production^30, 31^. Solid state synthesis of metal oxides from molecular precursor have several advantages over the other synthetic approaches as it is simple, gives good yields that facilitates large scale^32^.The use of ZnO as a photo-catalyst was studied in the degradation of Rhodamine B dye under UV radiation^33–35^. The effect of catalyst dose and particle size on the degradation efficiency of the dyes was studied^36^. In continuation to our previous work in synthesis and hybridization of metal oxides investigation and apply them as efficient materials in water treatment^37–42^ . ZnO mixed with ZnC were synthesized by benign solid state technique from citrate molecular precursor. Various techniques were used in characterization of the calcination products. The photocatalytic activity of the synthesized ZnO/ZnC mixture showed efficient photocatalytic activity in degradation of various dyes in comparison with other catalysts.

## Experimental

### Materials and methods

Zinc acetate dihydrate (Zn(CH_3_COO)_2_·2H_2_O ≥ 99%,Acros) and citric acid anhydrous (C_6_H_8_O ≥ 99.5%, Fisher scientific), Dyes: Rhodamine B ≥ 95% (HPLC), Merck) and Fluorescein sodium salt ≥ 97.5% zHPLC),Merck).The solvent used is deionized (DI) Milli-Q water. The UV–Vis absorption spectra of the prepared samples was measured using a double beam spectrophotometer (Cary 5000 UV–Vis-NIR, Agilent Technologies). The FTIR spectra were collected using a FTIR spectrometer (Vertex 70, Bruker, Germany). XRD of the as-prepared Zinc-Citrate precursor and ZnO samples were characterized using a Malvern Panalytical Empyrean 3 diffractometer. The morphology and particle size of the samples were determined by FESEM, (Quattro S, Thermo Scientific).

### Synthesis of zinc citrate precursor and ZnO nanoparticles (NPs)

The precursor was prepared by semisolid method^41^ in which, of Zn(CH_3_COO)_2_·2H_2_O and citric acid in (1:1), (1:2) and (1:3) molar ratio were grinded well in a mortar till a very fine mixture was obtained. Then 1 mL of Milli-Q-water was added with continuous grinding till a homogenous paste was formed. The paste was dried at 100 °C for 3 h. The calcination temperature has been determined from TGA of the precursor Fig. S1. Previous reports^43^ indicated the formation of sheets of ZnO when the precursor was calcined at 700 °C, which is important in photo-catalysis. In addition, the powder obtained from (1:2) molar ratio was calcined in air at 400, 500, 600 and 700 °C for 2 h at atmospheric pressure to investigate the impact of temperature on particle shape in Fig. S1 (400–600).

Anal. Found for Zn(C_6_H_7_O_7_)0.2H_2_O **C**, 24.0; **H**, 3.9; **Zn**, 23.3%. Calc.: **C**, 24.7; **H**, 3.7; **Zn**, 22.4%

### Photo-catalysis

To evaluate the photocatalytic activity of the synthesized C-ZnO, Rhodamine B (RB) and Flurocine (Flu) were utilized as models for resistant cationic and anionic organic water pollutants. The stock dye solution concentrations for RB and Flu were 5 × 10^–5^ and 6 × 10^–5^ M, respectively. A batch reactor containing the proper amount of photo catalyst (0.1 g) and the investigated dye solution (100 mL) was ultrasonically agitated for 60 s to ensure photo catalyst dispersion, and the suspension was magnetically stirred in the dark at 500 rpm for 60 min to ensure adsorption–desorption equilibrium. Then, the photo degradation tests were conducted using a 15 W Sylvania UV-A lamp for UV-A irradiation (wavelength 315–400 nm); the batch reactor was irradiated for 120 min with continuous stirring at 500 rpm; 5 mL-aliquots were pipetted out every 30 min during the irradiation process; and the aliquots were centrifuged for 30 min at 3300 rpm. The UV–Vis absorbance spectra of the filtrates were analyzed using a Thermoscientific Evolution 300 UV–Vis spectrophotometer, allowing the dye removal percentage to be calculated using Eq. (1):

$${\text{Removal}}\% \, = \,\left[ {\left( {{\text{A}}_{0} {-}{\text{A}}_{{\text{t}}} } \right)/{\text{A}}_{0} } \right]\, \times \,{1}00$$

where A_o_ and A_t_ are the absorbance of the investigated dye (RB or Flu) at λmax (554 nm for RB and 490 nm for Flu) in the dark and at a time (t) of irradiation, respectively.

## Results and discussions

### Synthesis of the precursor and ZnO-Nps

Optimized molar ratio (1:2) of Zn(OAc)_2_.2H_2_O : citric acid (CA) to form main precursor to achieve the thinnest thickness of Nano-sheets according to past reports^43, 44^ for photo catalysis applications. Prepared from the reaction of Zn(OAc)_2_.2H_2_O with citric acid (CA), it resulted [Zn(CA)0.2H_2_O] in a 1:1 molar ratio, and a residual of citric acid may remain unbound. FESEM of the precursor Fig. S2A. Indicates that, the precursor is a flakes of crystalline materials and some irregular granules. EDX indicates that the precursors include both Zn, C and O. The disappearance of any other elements confirms the purity of the precursor. The mapping of Zn, O, and C atoms indicates that the atoms are regularly distributed, and Zn atoms are surrounded by oxygen atoms as indicated from the magnified image of the mapping of the total distribution of elements and EDX analysis Fig. S2B–F. After Calcination at 700 °C for three precursors, the elemental analysis ratio obtained from Energy Dispersive X-Ray Analysis (EDX) indicated that the average of carbon weight percentage content in ZnO-nanoparticles obviously increased from 21.57, 34.5 and 40.9 for (1:1), (1:2) and (1:3). Figure S3 (1:1) and Fig. S3 (1:3) for (1:1) and (1:3) ratio respectively.

### Characterization of the precursor

IR spectra of the precursor Fig. S4 was compared carefully with that of the free citric acid to deduce the mode of chelation of citric acid. Citric acid has three carboxyl groups; two of them (1 and 2) are symmetric, so the spectrum of the citric acid exhibits two bands at 3494 and 3292 cm^−1^ due to ν(OH) of the three carboxyl groups. Another shoulder band is observed at 3224 cm^−1^ owing to the free (OH). In addition to that, two strong bands are observed at 1735 and 1703 owing to ν_as_ (COOH) of the protonated three carboxyl groups^37^. In the spectra of the precursor, two bands are observed at 3468 and 3382 cm^−1^ owing to two ν(OH) of the two carboxyl groups (1 and 3), while the band of the free (OH) group has disappeared. It is worth noting that the shoulder at 3492 cm^−1^ due to the presence of unreacted citric acid. Two strong bands are also observed, including a strong band at 1702 cm^−1^ in its position as in the free acid due to the starching vibrations ν as (COOH) of the protonated carboxyl group (3). The second band at 1628 cm^−1^ is attributed to ν as (COO-) of the deprotonated carboxyl group (1)^37–39^ This band is shifted to a lower wave number as a result of the coordination of this group with the Zn(II) ion. The difference between the asymmetric and symmetric groups that lies at 1443 cm^−1^ is 180 cm^−1^ indicating the monodentate nature of this group. The broad band at 3584 cm^−1^ is attributed to a coordinated water molecule. Two weak bands are observed at 510 and 525 cm^−1^ owing to Zn–O band^40^. From the above findings, it is suggested that CA chelates Zn(II) as indicated in Fig. S5.

Figure S6. Precursor XRD pattern shows peaks at 2θ = 11.0, 13.5, 15.8, 21.8, 26.5, 31.1° confirming the crystallinity nature of the precursors. The peaks assigned with asterisk point to crystalline citric acid the presence of these peaks point to unreacted citric acid.

### Characterization of the calcination products

#### XRD and XPS

**Figure 1:**
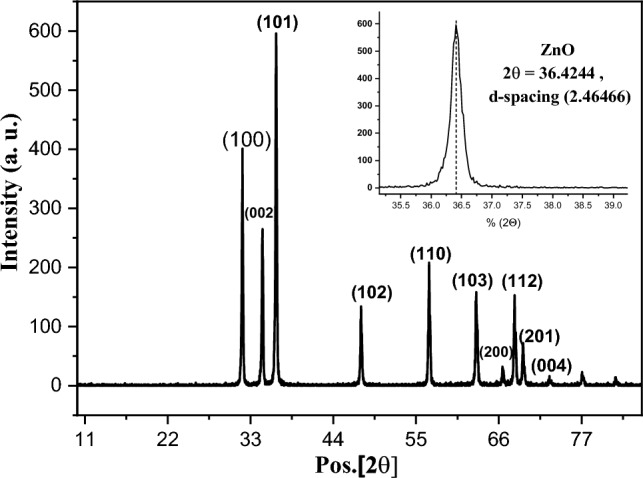
XRD pattern of ZnO nanoparticles.

XRD diffraction pattern of the product resulted from the calcination of the precursor Fig. 1. indicates diffraction peaks at 2θ = 31.9, 34.5, 36.4, 47.6, 56.7, 62.9, 66.4, 68.1, 69.2, 72.6 and 77.1o corresponds to the planes (100), (002), (101), (102), (110), (103), (200), (112), (201), (004) and (202). These peaks is well indexed to C-ZnO (JCPDS card NO. 01–075- 0576) with hexagonal structure, space group p63mc and lattice parameters a = b = 3.24 Å, c = 5.19 Å, α = β = 90° and ɣ = 120°.The determined crystallite size from the major peak at 36.4224 from Debye-Scherer relation^45^: D = 0.94 λ/β cosθ is 75 nm.

The careful observation of the XRD pattern indicates that the doped C has shifted the plane (101) to a higher 2θ, which leads to deformation in the unit cell^42^. The calculated crystallite size, lattice parameters, and unit cell volume of ZnO have changed in comparison with pure ZnO due to the change in the d-spacing tabulated in Table S1^42–44^. The C-doping effects on the reduction of the cell volume and the difference in the lattice parameters of C-ZnO can be attributed to the structure defects (O_vac_) caused by C-doping. The occupation of O_vac_ by the carbon anion with a radius (69–76 pm), which is higher than that of oxgen (57–66 pm), will lead to a disturbance in the lattice volume and parameters of ZnO. Also, due to the charge of both carbon and oxygen, the substitution of O(–II) by C(–IV) will unbalance the charge of the system, which requires oxygen loss to remain balanced. The results agree with previous research where C-doping-induced unit cell changes were also observed^45^. The presence of carbon was supported by the carbon weight ratio in XPS and EDAX results.

#### XPS

**Figure 2:**
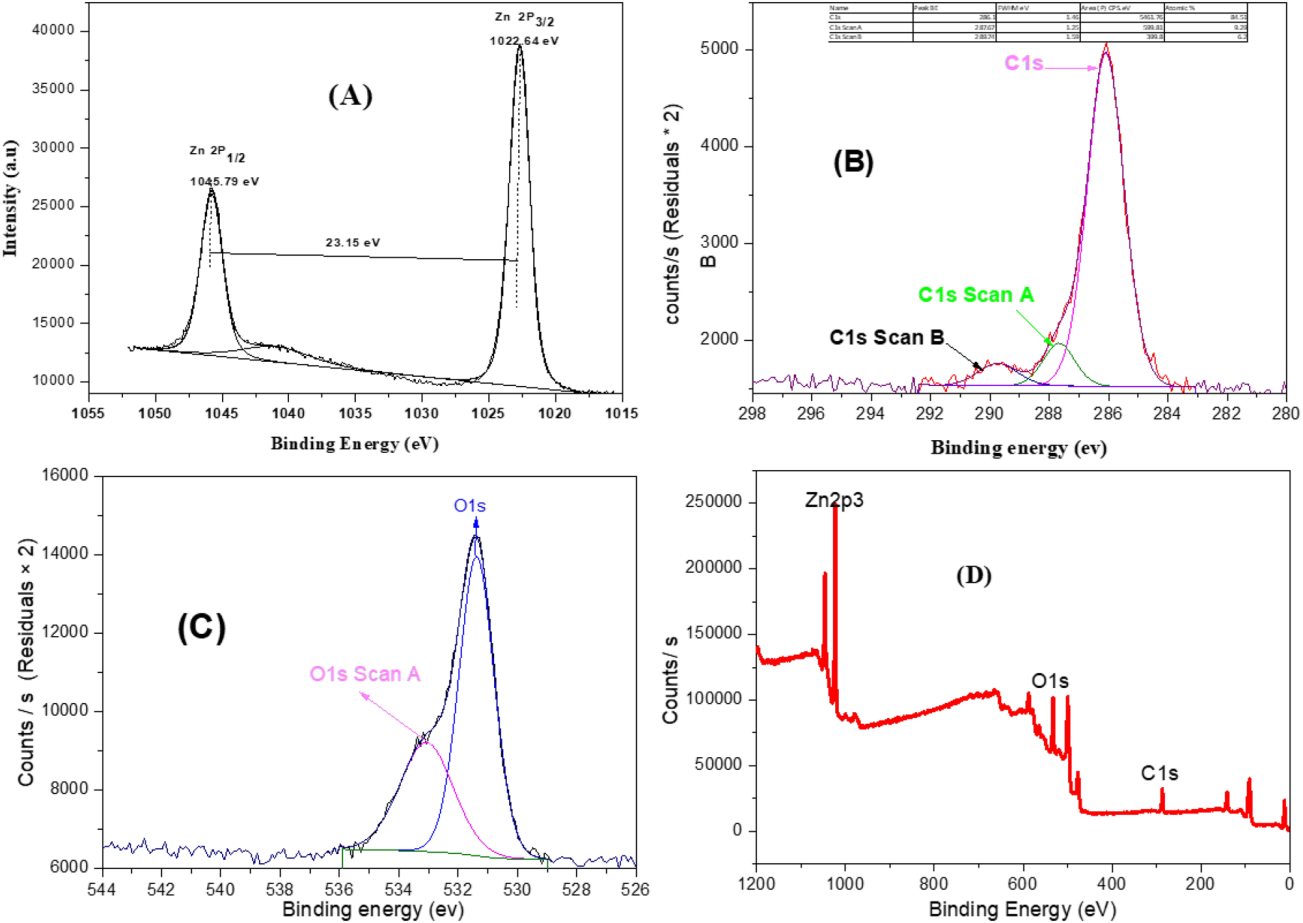
XPS of ZnO nanoparticles (**A**) Zn 2p spectrum (**B**) C 1 s specturm (**C**) O 1 s spectrum (**D**) XPS survey spectrum of ZnO nanoparticles.

Additionally, XPS was done to ensure the chemical composition of the tow dimentional C-ZnO surface. XPS survey spectra of Zn 2p and O 1 s of C-ZnO nanoparticles are shown, respectively, in Fig. 2A–D. The binding energies are calibrated considering the C 1 s emission centered at 284.5 eV. The C 1 s spectrum of doped ZnO can be deconvoluted into two components at 286.1, 287.6 and 289.7 eV. The atomic ratio of Zn, O, and C were 58.43, 38.61 and 2.96%, respectively. Zn 2p spectrum displays two main peaks of Zn 2p _1/2_ and Zn 2p _3/2_ states centered at 1022.64 eV and 1045.79 eV, respectively. These peaks confirm the presence of Zn atom in lattice of ZnO crystal 46. The difference in binding energies between the peak of Zn 2p _3/2_ and that of Zn 2P_1/2_ is 23.15 eV; that is the characteristic for C-ZnO. The peak profile of the O 1 s state exhibits a broad band that extends from 530 to 534 eV. The deconvusion of this peak exhibits two peaks: the first at 531.38 eV is attributed to lattice oxygen (Atomic % 64.26), and the second peak at 533.0 eV is owing to surface oxygen atoms (Atomic % 35.74)^23^. XPS indicates that the carbon composition is relatively high (34%), which may arise from the incomplete combustion of the precursor.

#### FT-IR and UV–visible spectra

**Figure 3:**
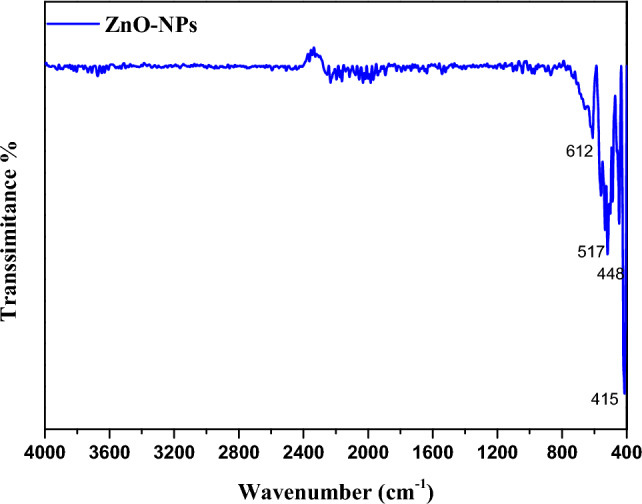
IR spectrum of ZnO NPs.

**Figure 4:**
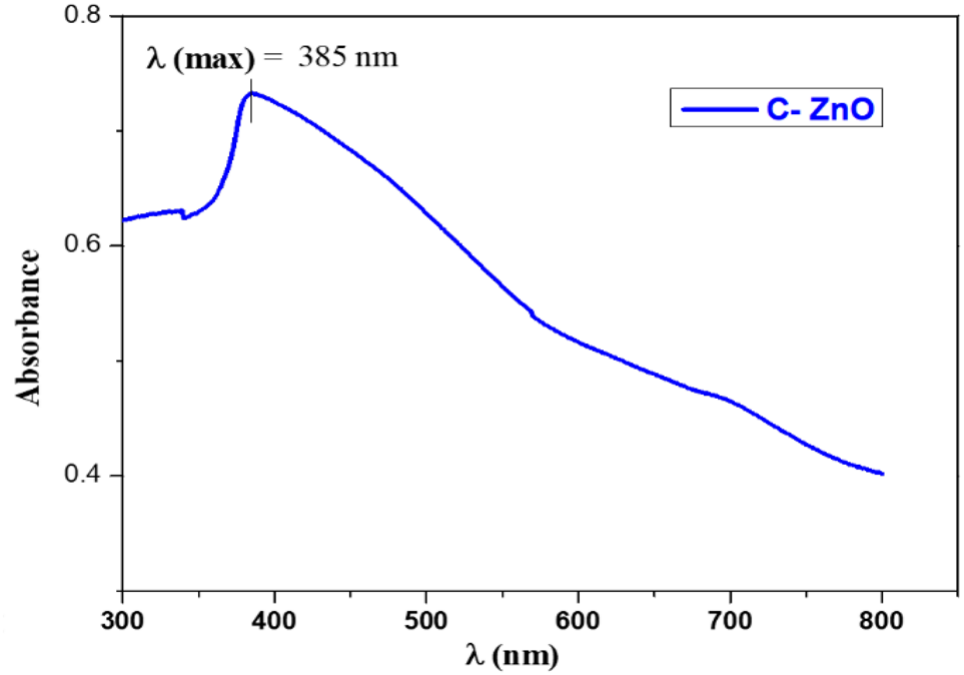
UV spectrum of ZnO NPs.

The formation of ZnO after calcinations of the precursor was further supported by IR and UV spectra. The IR spectrum of ZnO Fig. 3 shows bands at 415, 448, 517, and 612 cm^−1^, these bands are characteristic for ZnO nanoparticles^17^.Also, UV- spectrum Fig. 4 shows a characteristic band at 385 nm of C-ZnO that can be attributed to the intrinsic band-gap absorption of ZnO^46^.

#### FESEM

**Figure 5:**
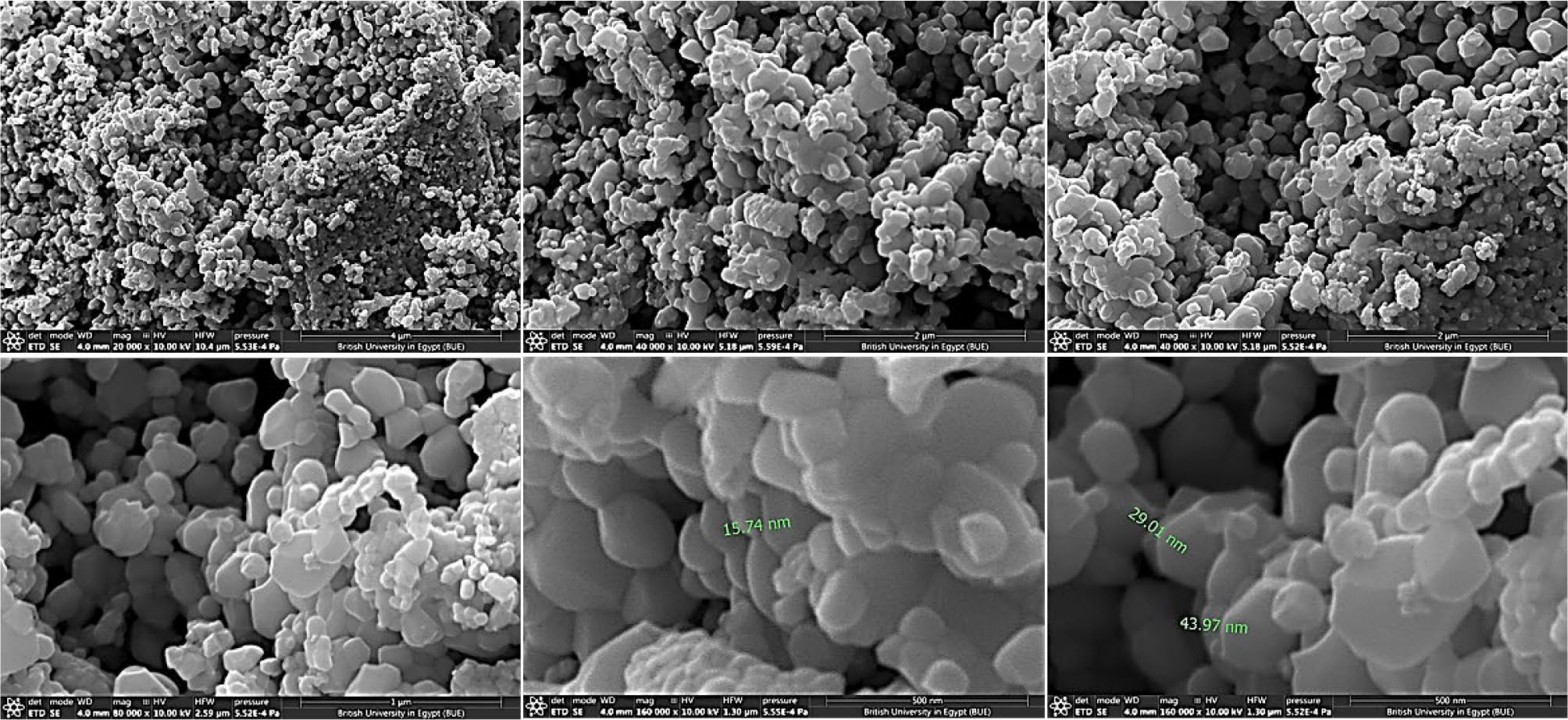
FESEM of ZnO NPs with different magnifications.

**Figure 6:**
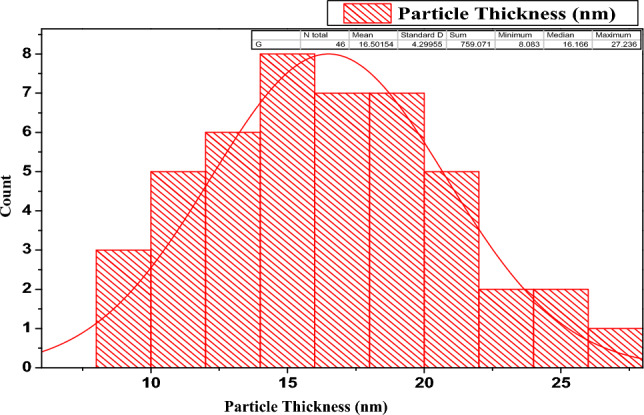
Particle thickness distribution.

**Figure 7:**
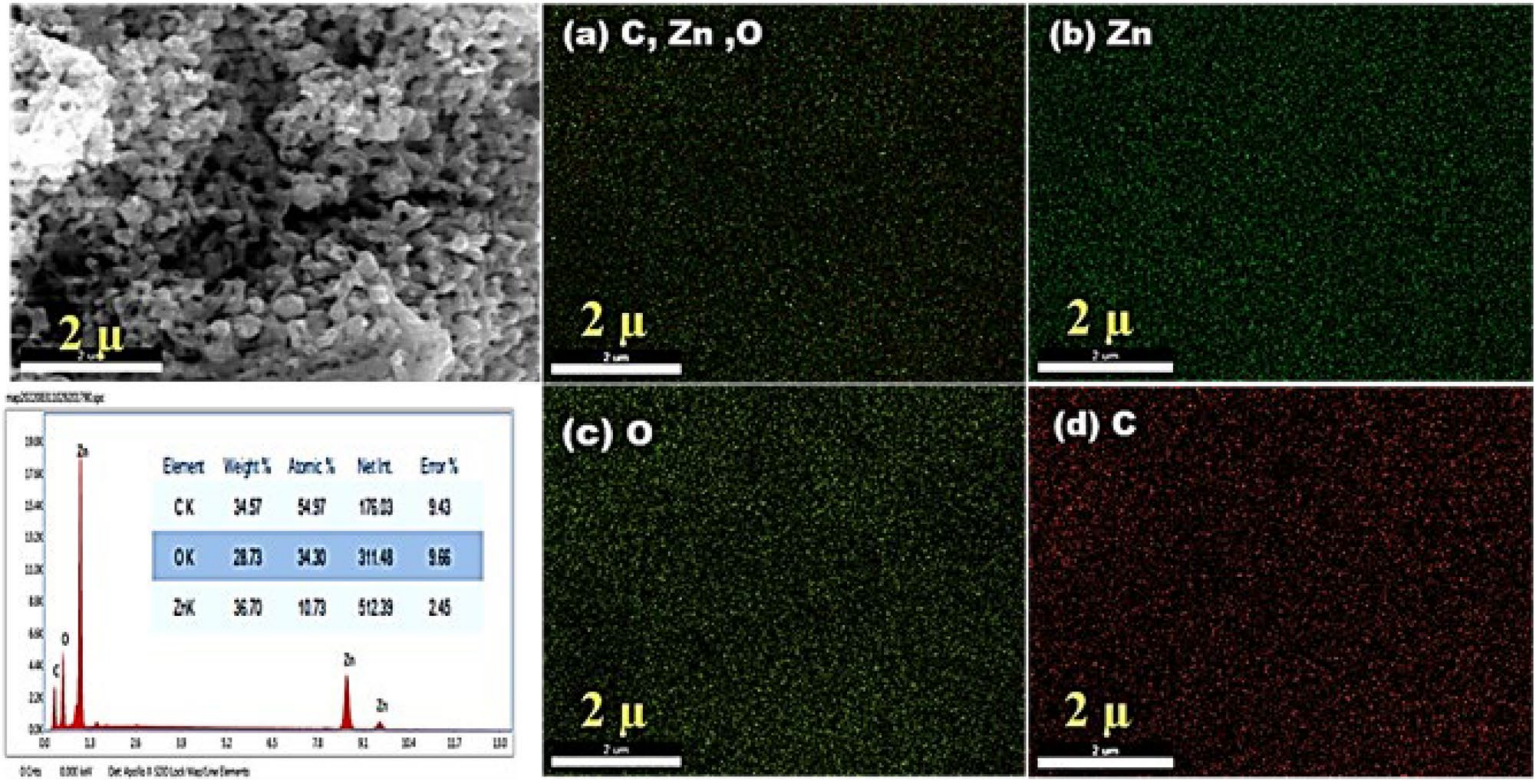
EDS for total element’s distribution. (**a**) Elemental mapping in total distribution of Zinc, Carbon and Oxygen elements, (**b**) Zn mapping, (**c**) Oxygen mapping and (**d**) Carbon mapping.

FESEM images Fig. 5 show aggregates of granules and irregular sheets with average particle size 33 nm and average thickness 25 nm Fig. 6. These crystalline sheets are arranged in layers. The mapping of ZnO nanoparticles Fig. 7 indicates that oxygen is regularly distributed around Zn atoms.

**Figure 8:**
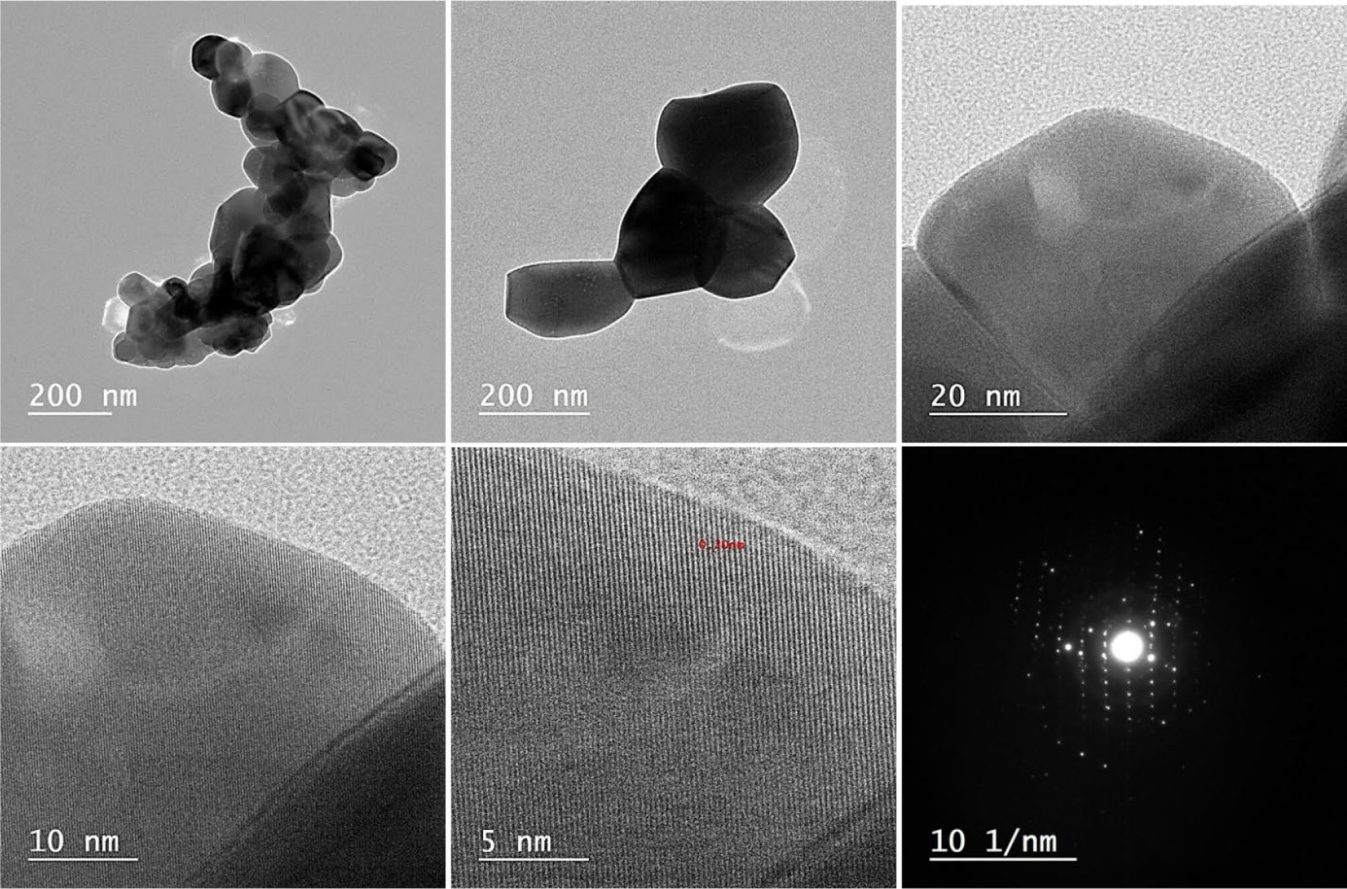
HR-TEM images at different magnification of ZnO NPs and SAED pattern.

**Figure 9:**
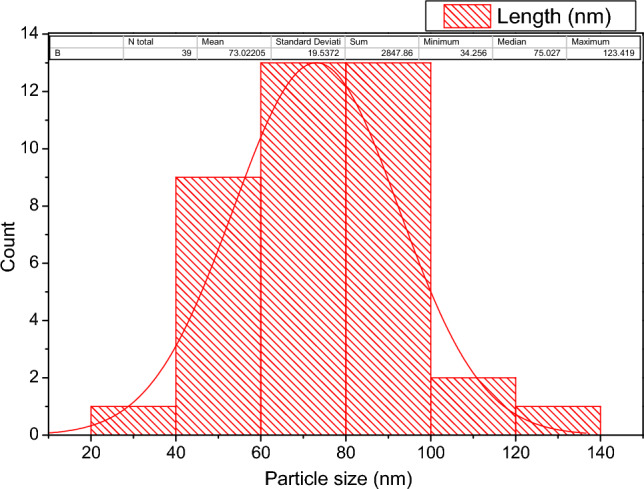
Histogram of particle size distribution.

Figure 8 displays the ZnO HR-TEM images and SAED. The SEM results are supported by the TEM images of ZnO, which show that the particles are virtually hexagonal with just a small thickness fluctuation. According to the histogram in Fig. 9, the range of particle sizes was 20–140 nm within an average of 73 nm. According to these images, the majority of ZnO NPs have hexagonal shapes and have an average particle size of 100 nm. The SAED pattern shows that the synthesized ZnO's diffraction rings displayed Debye–Scherrer rings with the designations (010), (002), (011), (012), (110), and (103), respectively. The TEM analysis estimates of particle size are comparable to the XRD analysis estimates; moreover, selected area electron diffraction (SAED) appears to be a good argument as a simple and convenient method for characterizing the macroscopic structures of 2D materials, and the instrument we constructed allows the study of the weak interaction with 2D materials^48^. SAED pattern of 2D material nature, which cannot show any high-order Laue zones since there are few layers in the beam direction. In comparison to HOLZ rings regarding 3D shape^49^ which obviously illuminated herenin and revealed layered 2D materials with low symmetry, 2D materials have emerged as anisotropic electronic and optoelectronic candidates.

### Optical properties

**Figure 10:**
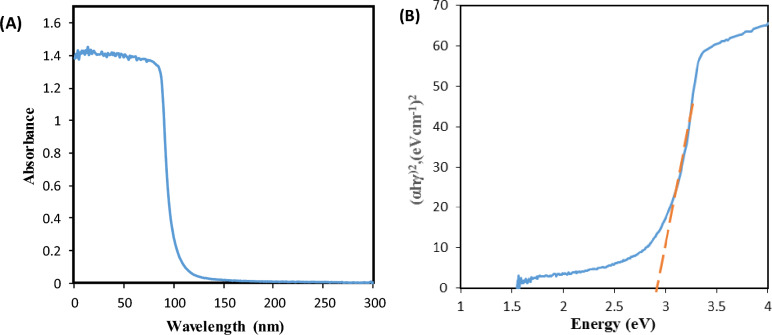
(**A**) UV–Vis diffuse reflectance spectrum and (**B**) Tauc plot of ZnO NPs.

For the purpose of displaying the absorption profile and optical characteristics of the nanoparticles, DRS is a necessary technique. The absorption-band-edge of ZnO nanoparticles is seen at 100 nm in Fig. 10A, which corresponds to a band gap energy of 2.9 eV (Tauc plot), in Fig. 10B The reduction of the optical band gap in comparison with the commercial (3.7 eV) may come from the resulted carbon from the incomplete combustion of the precursor^47–49^. The synthesised ZnO shows a shift in wavelength and a decrease in band gap, boosting its catalytic activity to the visible range Fig. 10.

### Photocatalytic activity

The photocatalytic activities of the synthesized ZnO NPs were evaluated via the photodegradation of both anionic and cationic dyes under UV irradiation. Fluorescein dye was used as a type of anionic dye, while Rhodamine B was the cationic dye. Prior to illumination, 100 mg of photocatalyst was added to the dye aqueous solution (100 mL, 10 ppm). The solution was stirred in the dark for 60 min in order to achieve absorption–desorption equilibrium, then the photocatalytic reaction was started. The photocatalyst will then be exposed to UV irradiations for the desired time.

**Figure 11:**
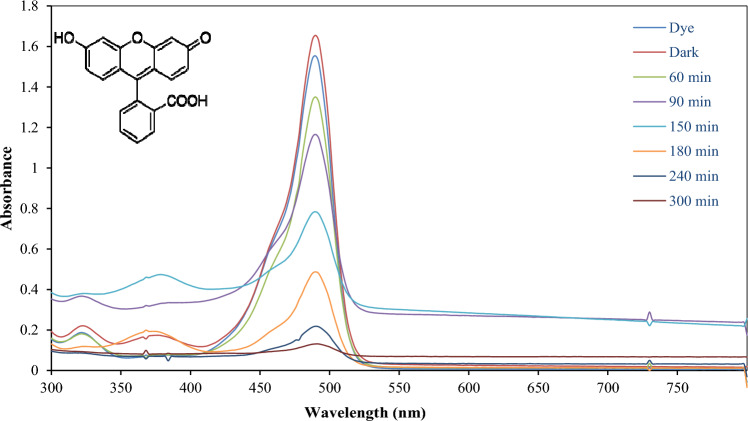
UV–visible spectra of Flu dye solution irradiated with UV light at different time intervals in presence of C doped ZnO photocatalyst.

Although bulk ZnO has barely low photocatalytic reactivity under UV irradiations due to the rapid recombination of the charge carriers and the wide band gap energy, it displays 99% degradation of Fluorescein dye after 240 min, as shown in Fig. 11.

**Figure 12:**
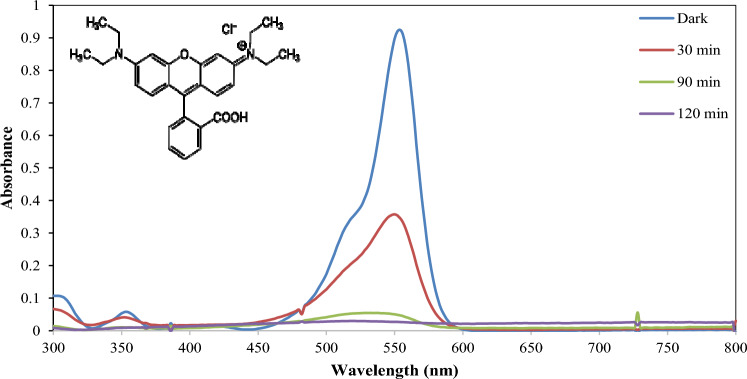
UV–visible spectra of RhB dye solution irradiated with UV light at different time intervals in presence of C doped ZnO photocatalyst.

Also, Fig. 12 displays the perfect photocatalytic behavior of prepared ZnO toward the photodegradation of Rhodamine B dye under UV irradiation sources; nearly complete decolorization was accomplished after only 120 min., of UV-A irradiations. And this give superiority of C-ZnO in Comparison over the photocatalytic activities of different ZnO doped catalysts toward the photo-degradation of RB in Table 1.

**Table 1:** Comparison between the photocatalytic activities of different ZnO doped catalysts toward the photo-degradation of RB.

Photocatalyst	Photocatalyst dose (g/L)	Time (Min)	% Photoegradation	References
ZnO/Cu0.5O	0.05	120	73.5	^50^
ZnO/In	0.5	120	76	^51^
ZnO/Mg	0.05	120	78.02	^52^
ZnO/Au	0.3	180	95	^53^
ZnO/Se	0.4	150	98.23	^54^
ZnO/Chl-Cu	0.03	120	99	^55^
ZnO/C	0.1	120	100	This work

**Figure 13:**
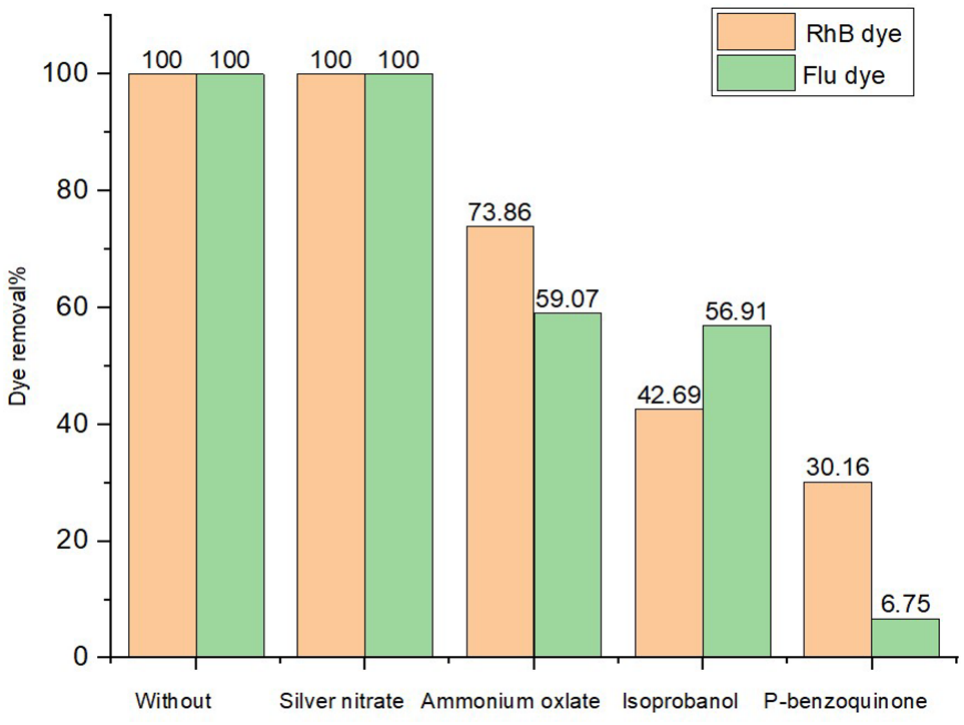
Effect of various scavengers over carbon-doped ZnO.

To investigate the role of reactive species in the degradation of RhB and Flu dyes, trapping experiments were done using ammonium oxalates, isopropanol, benzoquinone, and silver nitrate over a ZnO catalyst. The objective was to comprehend the roles played by positive holes, hydroxyl radicals, superoxide radicals, and the electron conduction band in the photodegradation process. It was observed that the addition of silver nitrate (AgNO_3_) had no effect on the efficiency of photodegradation, indicating that electron conduction has no effect on the removal of both dyes Fig. 13. The presence of benzoquinone, ammonium oxalate, and isopropanol had a significant effect on the photocatalytic performance of carbon-doped ZnO, indicating that hydroxyl radicals, positive holes, and superoxide radicals are involved in the degradation of RhD and Flu dyes. Regarding Flu dye, it is clear that superoxide radicals play the dominant role in the photodegradation process.

**Figure 14:**
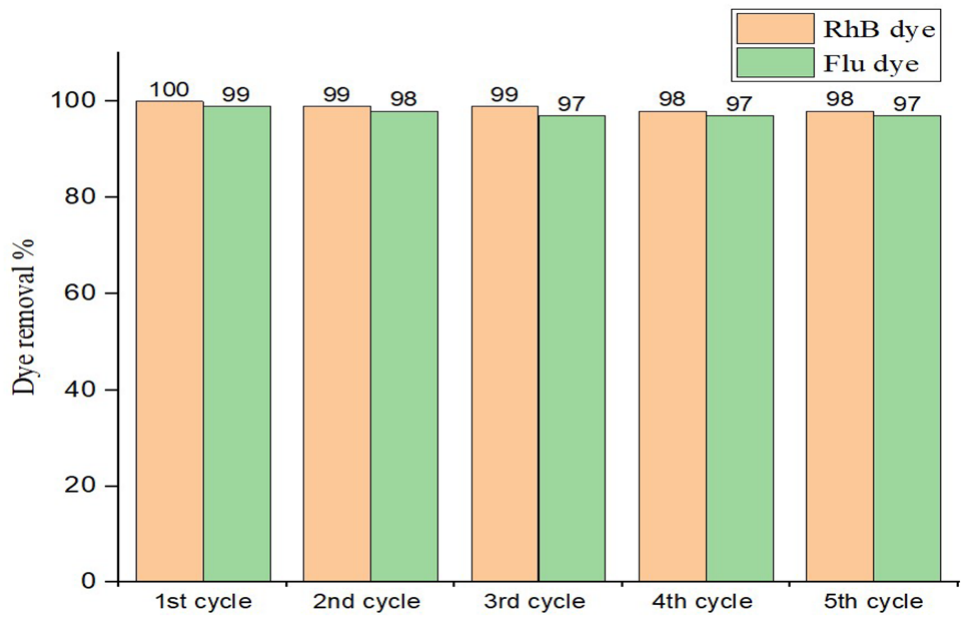
Cycling experiments of carbon-doped ZnO for RhB and Flu dye degradation under UV light irradiation.

Moreover, the high stability of the ZnO catalyst was demonstrated by the successful removal of RhB and Flu dyes even after five consecutive cycles Fig. 14 indicating that the prepared ZnO is extremely stable.

### Mechanism of photocatalysis

The mechanism for photocatalytic degradation of the used dyes onto ZnO nanoparticles by UV irradiation suggests the transfer of the electrons that exist in the valence band of ZnO to the conduction band under the effect of UV radiations. The absorbed energy should be higher than the current energy band gap of ZnO NPs (3.0 eV). The absorbed radiation will promote the electrons (e^−^) to the conduction band and holes (h^+^) in the valance band. The generated holes can oxidize the dyes directly or react with H2O generating hydroxyl radicals (·OH). On the other hand, the photoelectrons in the conduction band can reduce the adsorbed O2 on the surface of C-ZnO into superoxide radical (O^2−^). Both OH and·O^2–^ can decompose the dyes^56, 57^.

The suggested mechanism can be represented as follow:
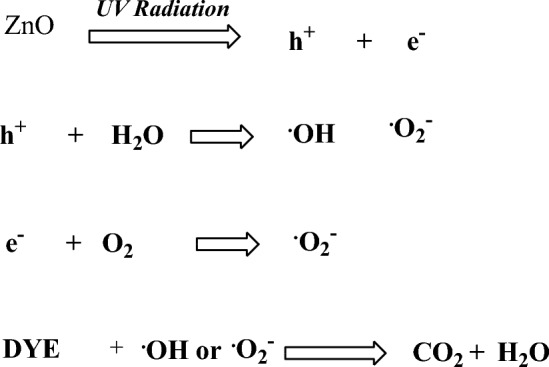


## Conclusion

ZnO nanoparticles have been synthesized by a simple solid state decomposition method. The used technique has advantageous as it is simple benign and provide high yield of 2D-carbon doped ZnO nanoparticles. The obtained ZnO have high photocatalytic activity in decomposing both cationic and anionic harmful azo dyes. The current work can be applied to synthesize other carbon doped metal oxides in two-dimensional scale that can be applied as catalysts in degrading and treatment of industrial water from harmful dyes.

### Supplementary Information

Supplementary Information.

## Data Availability

Data will be made available on request in contact to Prof.Medhat through (medahmed6@yahoo.com).
