# Supplementary material for: ZnO doped C: Facile synthesis, characterization and photocatalytic degradation of dyes
Source: Sci Rep. 2023 Aug 30;13:14173. doi: 10.1038/s41598-023-41106-4 (PMC10468539; doi:10.1038/s41598-023-41106-4)
Supplement: Supplementary file 1 — Supplementary Information. [file 41598_2023_41106_MOESM1_ESM.pdf]

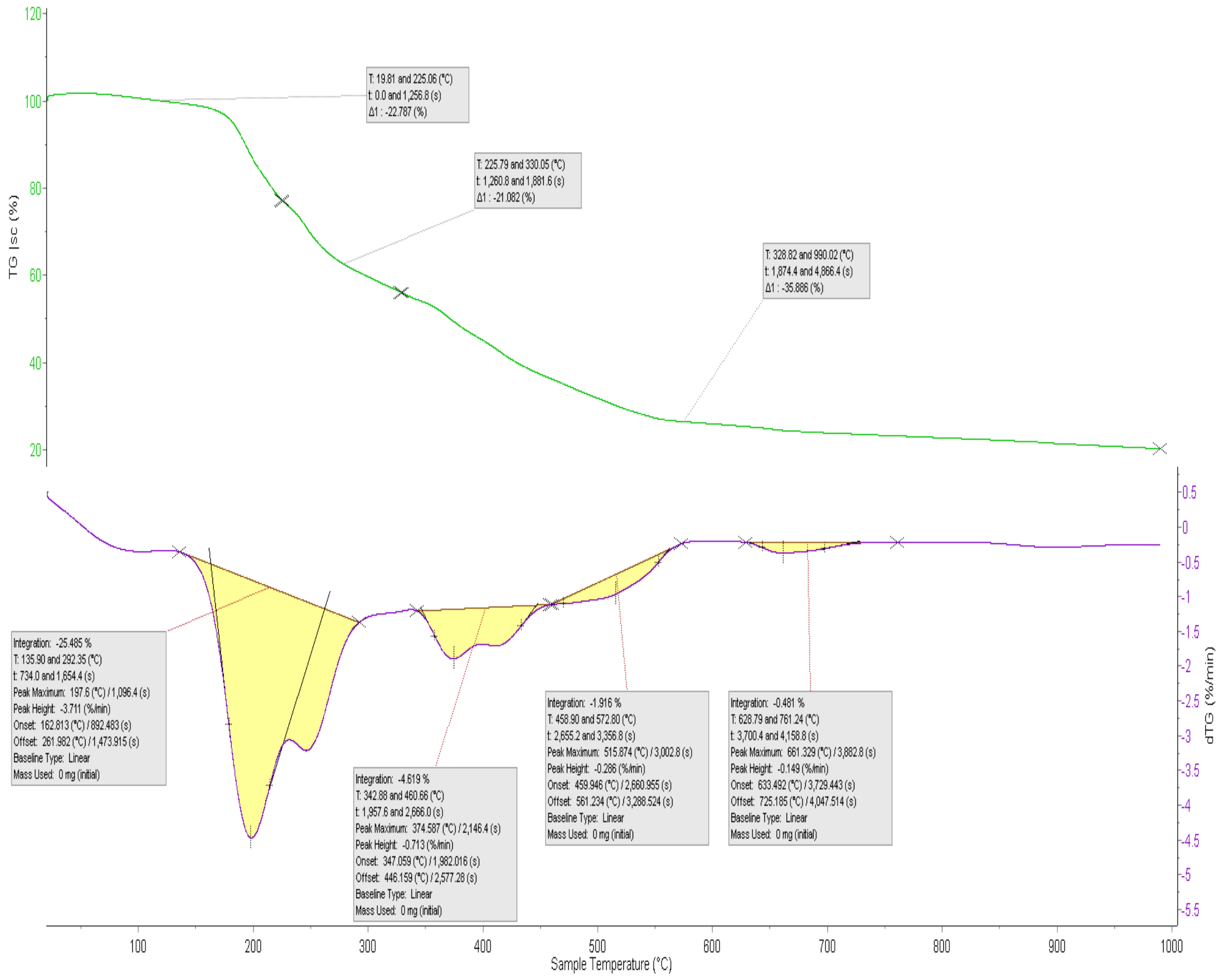

Fig. S1. TGA of Zn Citrate precursor

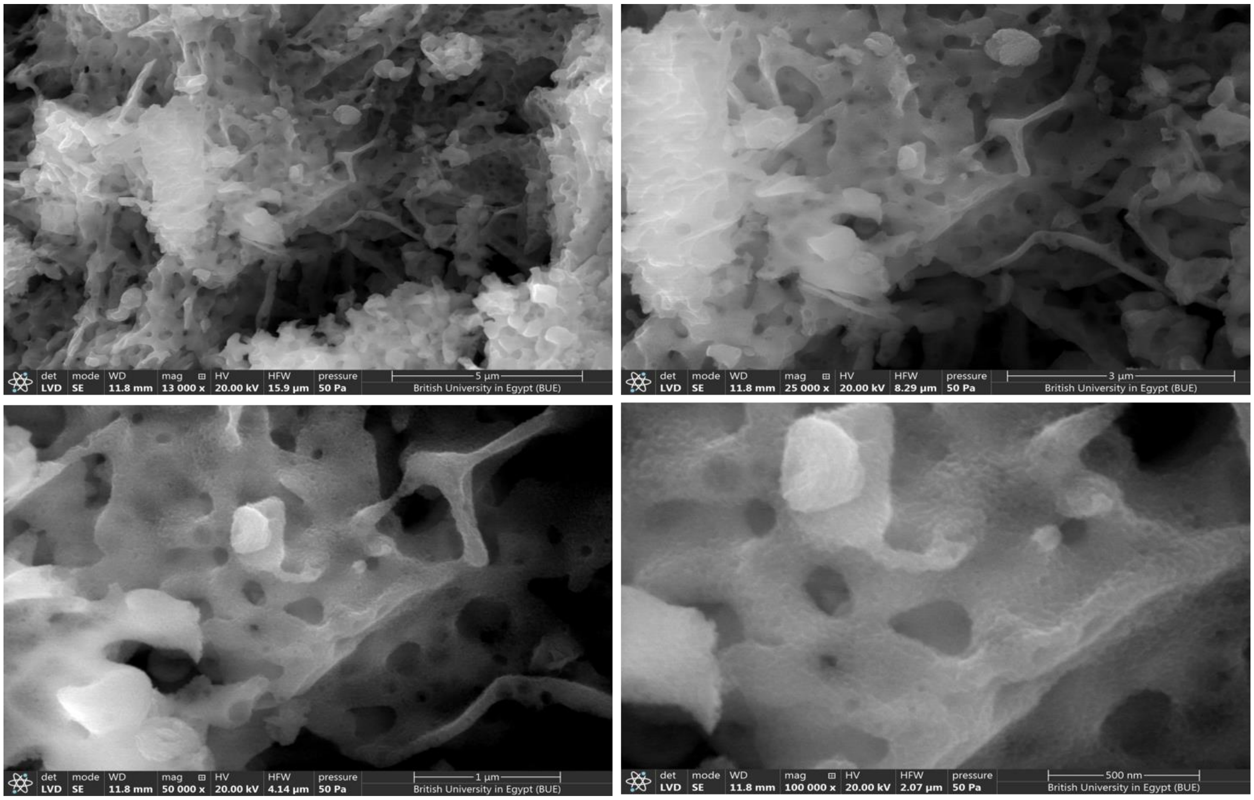

Fig.S1.(400) ZnO Nano-particles morphology after calcination at 400°C for 2 H at different scale Appears an similar morphologies with elastic agglomeration may due to residual of organic moiety and this is consistent with results obtained from TGA-Chart .

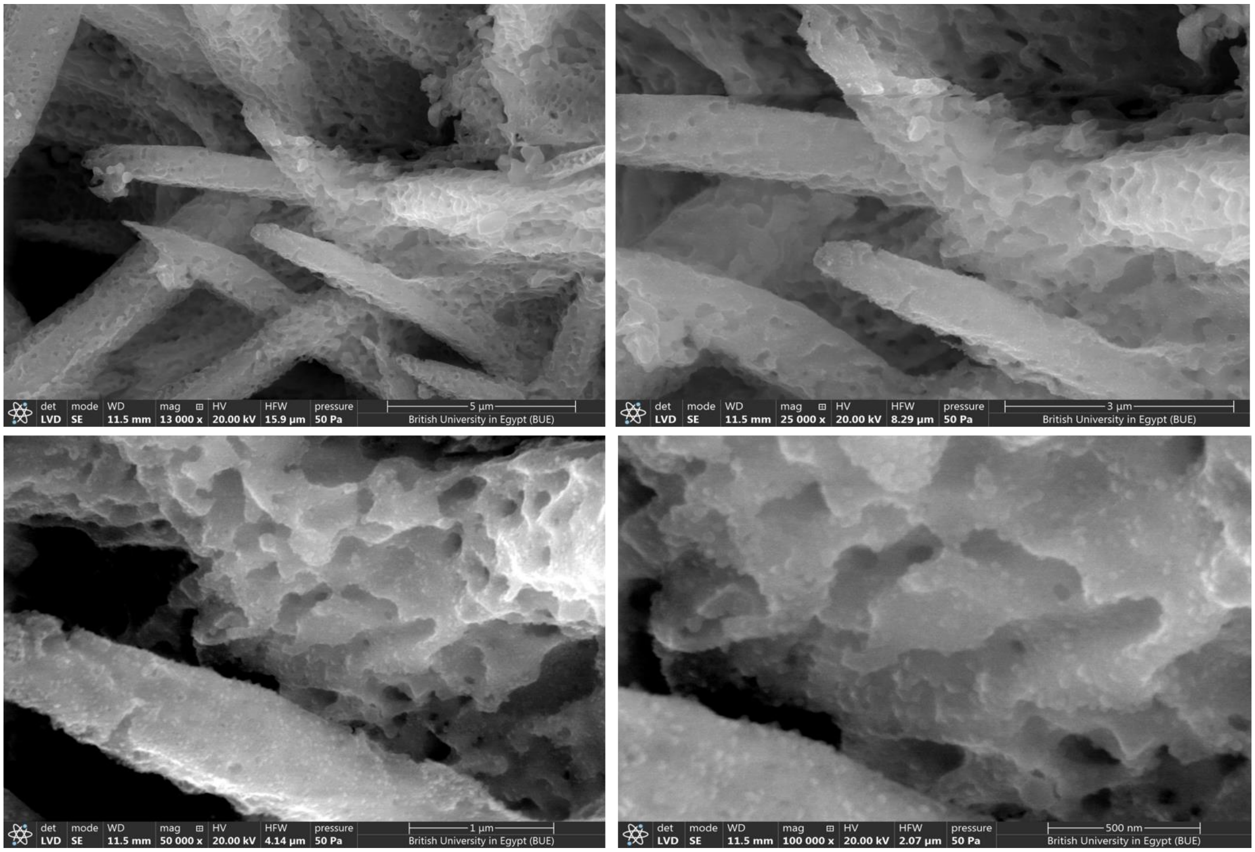

Fig.S1.(500) Zno Nano-particles morphology after calcination at 500°C for 2H at different scale Appears rods morphology with different length and diameter.

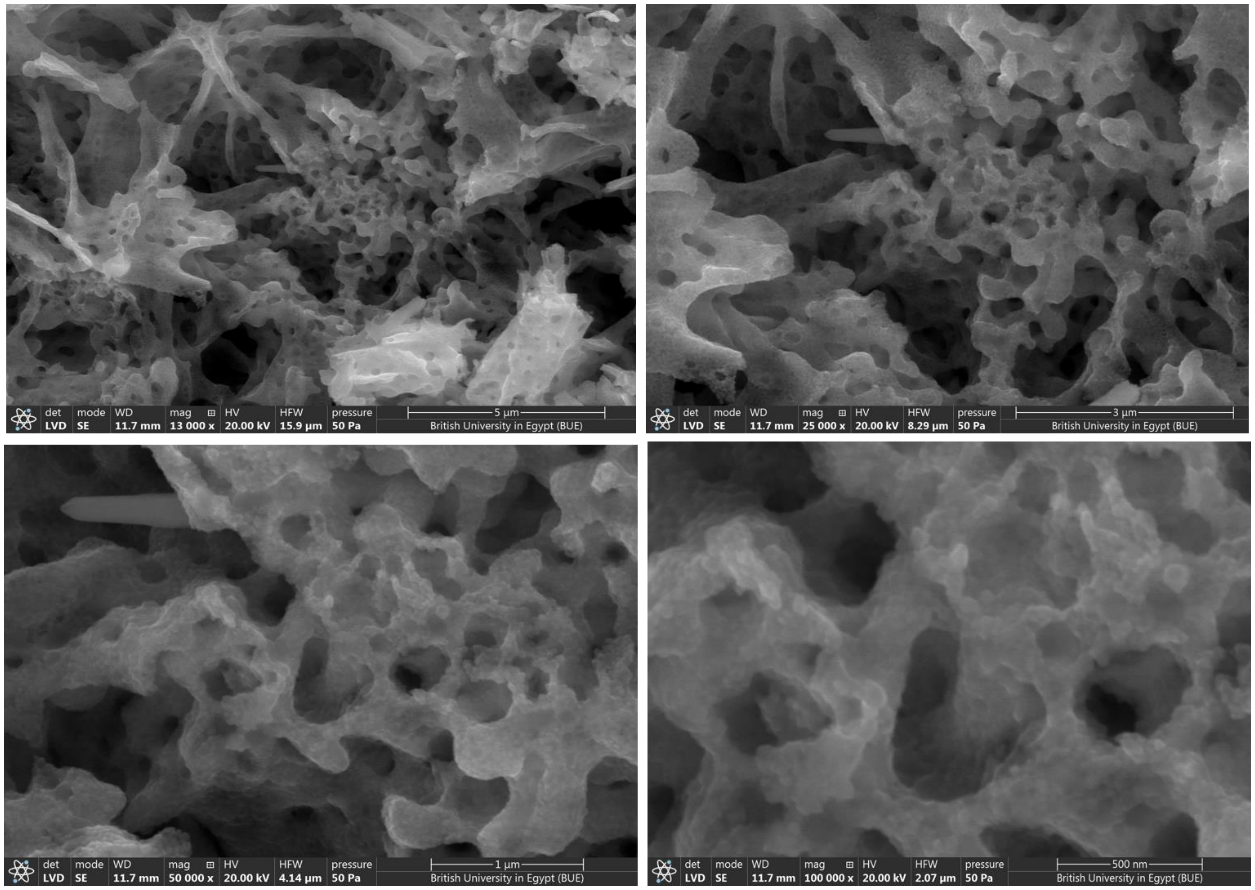

Fig.S1.(600) Zno Nano-particles morphology after calcination at $600^{o}C$ for 2H at different scale Appear small micro sheets stacking to each other forming clusters with macro-pores due to gases evolutions during calcination[1]

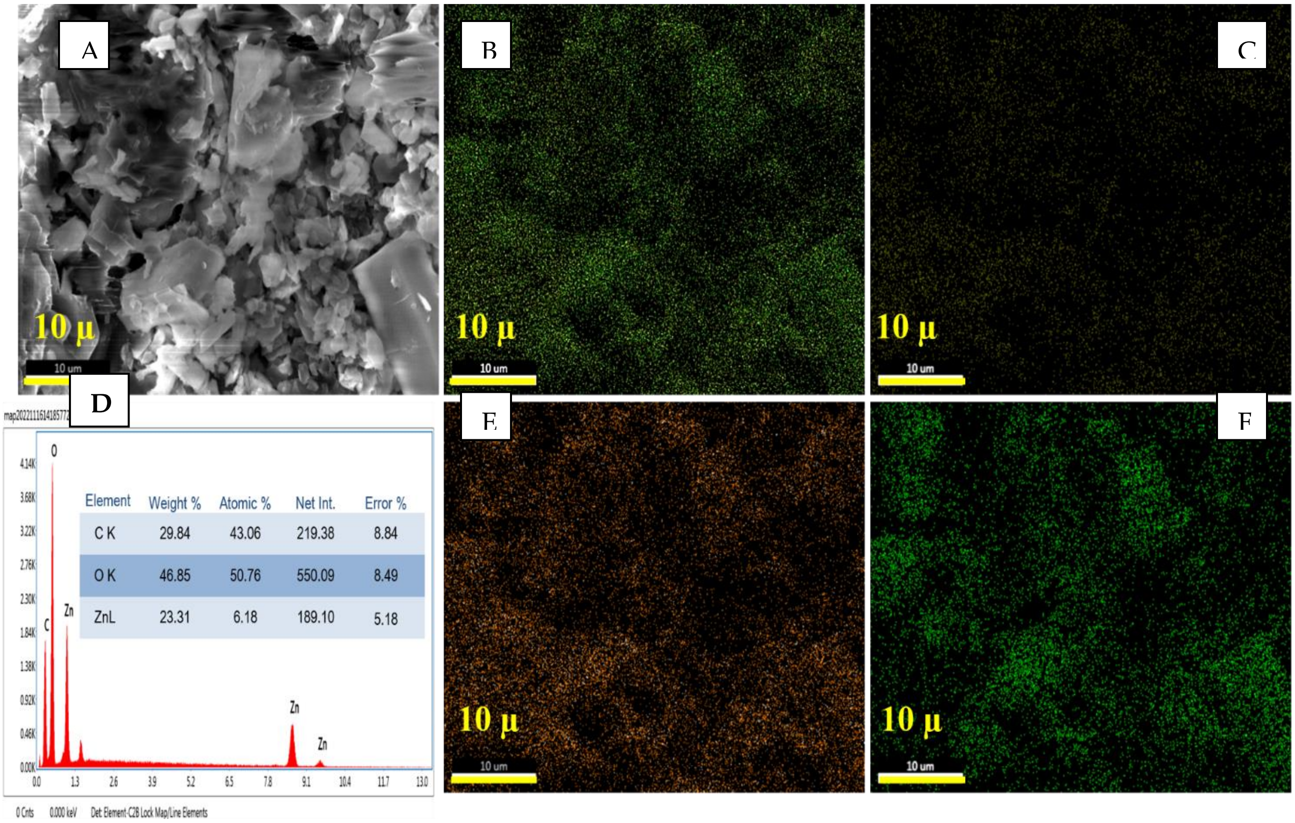

Fig S2. (A) FESEM of the precursor , **(B)** elemental mapping in Total distribution of Zinc, Carbon and Oxygen elements , **(C)** Carbon Mapping , **(D)** Energy Dispersive X-Ray Analysis (EDX) , **(E)** oxygen mapping and **(F)** Zinc Mapping .

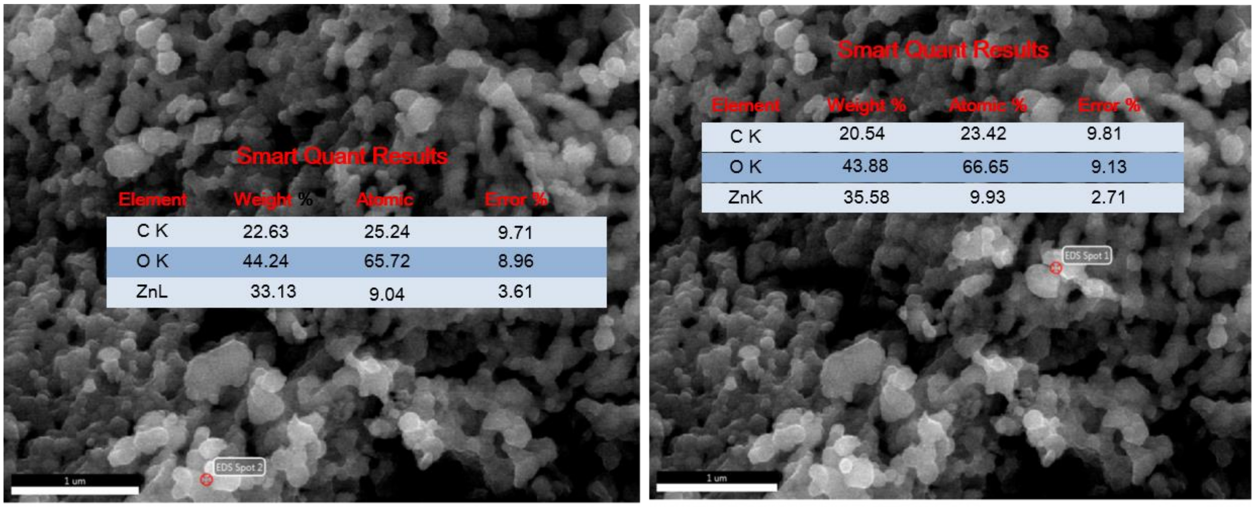

Fig S3.(1:1). Elemental ratio obtained from Energy Dispersive X-Ray Analysis (EDX) indicated that average of carbon weight % content in ZnO-Nanoparticles synthesized from (1:1) precursor is 21.57 % .

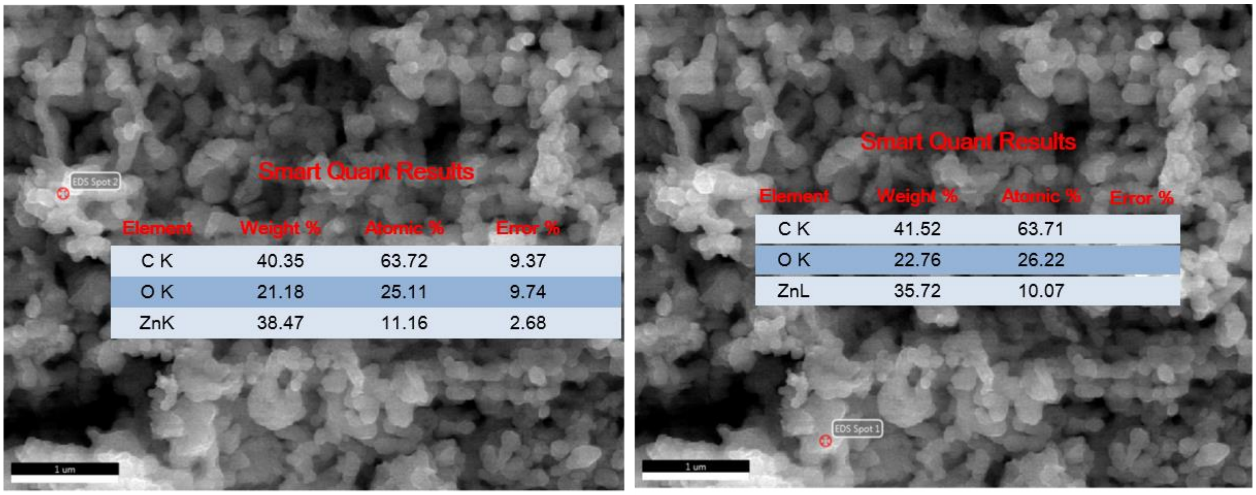

Fig S3. (1:3). Elemental ratio obtained from Energy Dispersive X-Ray Analysis (EDX) indicated that average of carbon weight % content in ZnO-Nanoparticles synthesized from (1:3) precursor is 40.9 %.

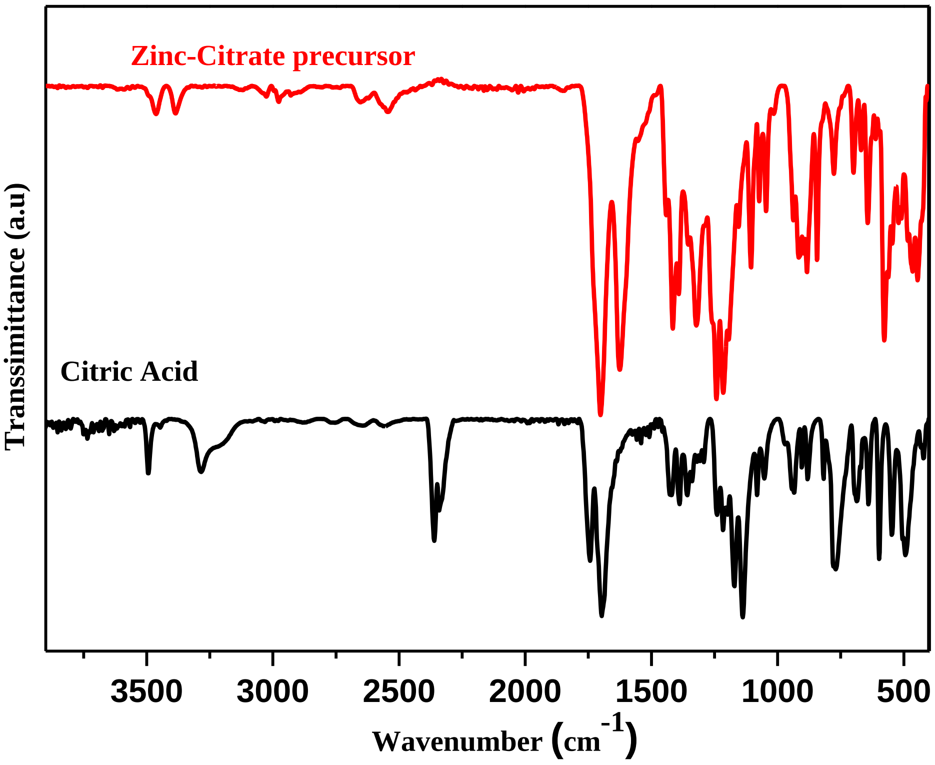

Fig. S4. IR spectra of citric acid and Zn (citrate) precursor

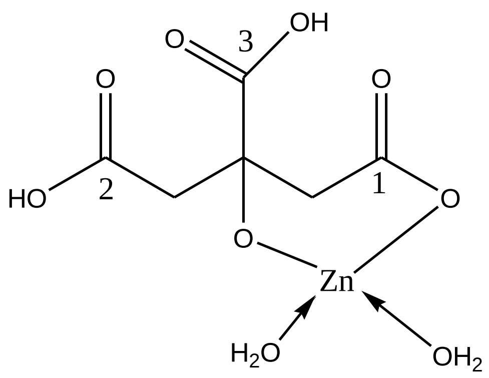

Fig. S5. Suggested structure of the precursor

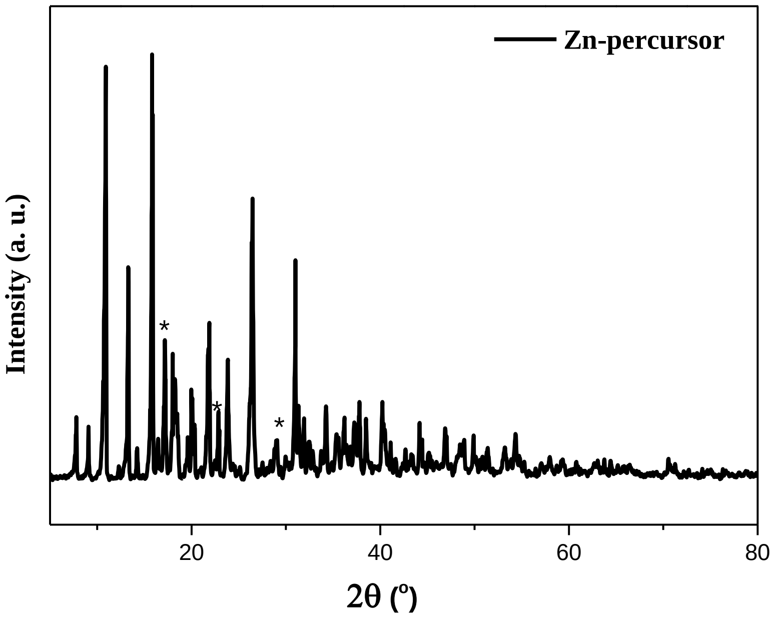

Fig. S6. XRD of Zn Citrate precursor

Table S1. XRD Crystal -Parametrs of ZnO and C-doped ZnO

ZnO

Rel. Int. [%]	d-spacing [Å]	Pos. [°2θ]
55.5	2.80826	31.840
41.3	2.59740	34.503
100	2.47039	36.337
21.45	1.90683	47.653
34.69	1.62098	56.7457
28.2	1.47393	63.016
4.3	1.40413	66.541
23.6	1.37538	68.120
11.6	1.35549	69.261
1.9	1.29870	72.759
3.7	1.23520	77.162
1.9	1.17875	81.610
7.5	1.09063	89.867

C-doped Zinc Oxide

Rel. Int. [%]	d-spacing [Å]	Pos. [°2θ]
66.88	2.79897	31.9489
41.77	2.59187	34.5788
100	2.46466	36.4244
21.45	1.90557	47.6865
34.69	1.62098	56.7457
26.9	1.47442	62.9922
5.96	1.40494	66.498
25.63	1.37592	68.0899
11.11	1.35627	69.2151
1.98	1.29976	72.6905
3.48	1.23667	77.0536
1.74	1.18018	81.4901
8.46	1.09168	89.7575
